# Stress and its impact on healthcare professionals: A study on prevalence and coping strategies

**DOI:** 10.1192/j.eurpsy.2024.1255

**Published:** 2024-08-27

**Authors:** M. A. Lafraxo, H. Guider, Z. Boumaaize, H. Darif, Y. El Madhi, A. Soulaymani, H. Hami

**Affiliations:** ^1^Laboratory of Biology and Health, Faculty of Sciences, Ibn Tofail University, Kenitra; ^2^ Scientific Institute, Mohammed V University; ^3^Regional Center for Education and Training Professions, Rabat, Morocco

## Abstract

**Introduction:**

Stress is a significant issue among healthcare professionals and impacts both their personal well-being and the quality of care they provide.

**Objectives:**

This study evaluated the prevalence of stress among healthcare professionals and investigated the possible effect of physical activity on perceived stress levels.

**Methods:**

In 2019, a cross-sectional observational study of 30 nurses was conducted at the Hassan II Oncology Center in Oujda. A self-administered survey was used to gather information regarding the participants’ sociodemographic and professional characteristics. The Perceived Stress Scale was employed to gauge stress levels, whereas the Ricci-Gagnon questionnaire was used to determine physical activity levels and engagement in sports.

**Results:**

The study findings indicate that the sample had moderate levels of stress measured by the Perceived Stress Scale (PSS) and struggled with managing stress in diverse situations. Physical activity was common among 87% of the participants, as indicated by the Ricci-Gagnon questionnaire. In addition, a statistically significant correlation was found between stress levels and family situation (p = 0.05). The Perceived Stress Scale and the Ricci-Gagnon questionnaire exhibited high internal consistency, with Cronbach’s alpha values of 0.79 and 0.64, respectively.

**Conclusions:**

The study results have raised significant concerns regarding the effectiveness of different coping strategies in managing stress. In particular, the results indicate that engagement in physical activity and sports does not significantly affect stress levels. Thus, stress management training is recommended as the best strategy for stress prevention.

**Disclosure of Interest:**

None Declared

